# European study of frequency of participation of adolescents with and without cerebral palsy

**DOI:** 10.1016/j.ejpn.2013.12.003

**Published:** 2014-05

**Authors:** Susan I. Michelsen, Esben M. Flachs, Mogens T. Damsgaard, Jacqueline Parkes, Kathryn Parkinson, Marion Rapp, Catherine Arnaud, Malin Nystrand, Allan Colver, Jerome Fauconnier, Heather O. Dickinson, Marco Marcelli, Peter Uldall

**Affiliations:** a National Institute of Public Health, University of Southern Denmark, Oster Farimagsgade 5A, 1353 Copenhagen, Denmark; b School of Nursing & Midwifery, Queen's University Belfast, Medical Biology Centre, 97 Lisburn Road, Belfast BT9 5BN, Ireland; c Institute of Health and Society, Newcastle University, Royal Victoria Infirmary, Newcastle upon Tyne NE1 4LP, UK; d Klinik für Kinder und Jugendmedizin, Universitätsklinikum Schleswig-Holstein, Ratzeburger Allee 160, Lübeck 23538, Germany; e INSERM, U. 1027, Paul-Sabatier University, Toulouse, France; f The Queen Silvia Children's Hospital, Göteborg University, Göteborg S-41685, Sweden; g SIIM-Pole Exploitation, Université Joseph Fournier, CHU de Grenoble BP 217, Grenoble cedex 9 38043, France; h Azienda Sanitaria Locale Viterbo, Vi Enrico Ferri 15, Viterbo 01100, Italy; i Peter Uldall, Copenhagen University Hospital, Pediatric Clinic 1, Blegdamsvej 9, 2100 Copenhagen, Denmark

**Keywords:** Cerebral palsy, Disability, Impairment, Adolescence, Participation

## Abstract

Children with cerebral palsy participate less in everyday activities than children in the general populations. During adolescence, rapid physical and psychological changes occur which may be more difficult for adolescents with impairments.

Within the European SPARCLE project we measured frequency of participation of adolescents with cerebral palsy by administering the Questionnaire of Young People's Participation to 667 adolescents with cerebral palsy or their parents from nine European regions and to 4666 adolescents from the corresponding general populations. Domains and single items were analysed using respectively linear and logistic regression.

Adolescents with cerebral palsy spent less time with friends and had less autonomy in their daily life than adolescents in the general populations. Adolescents with cerebral palsy participated much less in sport but played electronic games at least as often as adolescents in the general populations. Severity of motor and intellectual impairment had a significant impact on frequency of participation, the more severely impaired being more disadvantaged. Adolescents with an only slight impairment participated in some domains as often as adolescents in the general populations. Regional variation existed. For example adolescents with cerebral palsy in central Italy were most disadvantaged according to decisional autonomy, while adolescents with cerebral palsy in east Denmark and northern England played sports as often as their general populations.

Participation is an important health outcome. Personal and environmental predictors of participation of adolescents with cerebral palsy need to be identified in order to design interventions directed to such predictors; and in order to inform the content of services.

## 1 Introduction

Social and physical participation and deciding how to spend your life are important for all people, including children and adolescents with and without disabilities. Participation is defined by the ICF–CY^1^ as ‘involvement in life situations’ but is nevertheless still being refined in terms of conceptualisation and measurement.^2–5^ It is generally regarded as consisting of components such as school life, family and peer group activities and engagement in work and leisure. Participation is amenable to intervention and is an important health outcome for intervention research.

Children with cerebral palsy (CP) aged 8–12 years participate less in everyday activities than children in the general populations.^6,7^ Participation of children with CP varies considerably between countries.^8^ The environment of children with CP also varies considerably between countries^9^ and higher participation is known to be associated with the availability of a facilitatory environment.^10^

Adolescence is a critical developmental period that forms the basis of social integration in adulthood. Change and adjustment may be more difficult for adolescents with impairments and may result in reduced adult participation. Indeed, adults with CP are disadvantaged according to employment and cohabitation.^11–14^

Few epidemiological studies have focused on participation of adolescents with CP and suitable measurement instruments are lacking.^15,16^ Participation has a variety of dimensions; some instruments to measure participation capture whether or not the individual participates in an activity and if so the level of difficulty experienced,^17^ while others measure frequency of or enjoyment with participation.^18^ Comparative studies of frequency of participation in adolescents with and without impairment may yield insights into where further work is needed to equalise these groups.

There is a lower frequency of participation among adolescents with CP, compared with adolescents without CP.^19–21^ Severity of impairments is seldom taken into account and if so only motor impairment is considered.^21,22^ Adolescents unable to self-complete are often excluded.^20,23^ In addition many studies target only specific areas of participation, for example leisure or physical activities^24^ or include younger children and do not ask about typical adolescent activities like online communication or spending time with a boy- or girlfriend.^21^

Studying inter-country levels of participation in adolescence has the potential to identify regions with more or less facilitatory environments. This paper aims to compare frequency of participation in everyday life of adolescents across the spectrum of severity of CP and adolescents in the general population in nine European regions. We use QYPP (Questionnaire of Young Peoples Participation) – a new instrument of frequency of participation capturing participation in typical adolescent activities at home, school or work, and during leisure.^16^ It was developed by interviews with adolescents with and without CP as well as with parents of adolescents with CP not able to self-complete.

## 2 Method

This study is part of the European multicentre SPARCLE study which examines the quality of life and participation of children and adolescents with cerebral palsy. Full details are published^25,26^ and key elements are summarised below.

Eight European regions with population-based registers of children with CP participated: north England, Northern Ireland, southwest Ireland, southwest France, southeast France, central Italy, west Sweden and east Denmark. A further region in northwest Germany recruited children from multiple sources; their age, gender, and levels of impairment were similar to those of children in the population-based registers, although German adolescents were interviewed at a slightly younger age.^27,28^

### 2.1 Participants

Children with CP, born 1991–97, were randomly sampled from the registers. The 818 children who entered SPARCLE1 were followed up in 2009/2010 aged 13–17 years; 594 (73%) agreed to participate and the overall participation rate from sampling in registries to follow-up in adolescence was 51%. In order to maintain statistical power for cross-sectional analyses and possible follow-up to adulthood, SPARCLE2 additionally sampled from adolescents who were eligible for SPARCLE1 but who had not participated in SPARCLE1; 73 agreed to participate. Hence 667 adolescents were included in SPARCLE2 and their characteristics are shown in Table 1. Only cross-sectional data from adolescents are analysed in this paper.

For comparison, adolescents in the same age range as those in SPARCLE2 were recruited from the general populations from schools in the uptake area of each cerebral palsy register. Schools were randomly sampled from lists of all schools in the areas. In total 52 schools (4666 adolescents) participated. Recruitment of schools, response rates and characteristics of adolescents in the general populations are shown in Table 2.

### 2.2 Measure of frequency of participation

The Questionnaire of Young Peoples Participation (QYPP) was developed in the UK, based on interviews with adolescents with CP,^15,16^ in part to enable comparison of frequency of participation in adolescents with and without CP. We used a preliminary short form of the questionnaire with 31 items (QYPP-SF) in SPARCLE2, since the final short version was not available at time of data collection for SPARCLE2. The final short version of QYPP has recently been published.^16^ The QYPP-SF was translated according to international guidelines.^29^ Most items ask how many times a day, week, month or year the adolescents participate, using discrete categories for responses. Three items describe how often the adolescent decide on different aspects of everyday life and have response options of “always”, “mostly”, “sometimes”, “seldom” and “never”.

### 2.3 Measure of severity of impairment

Adolescents with CP were classified into four groups of severity that took account of both walking ability and the presence of intellectual impairment defined as IQ < 70 (Table 1). Severity of impairment was assessed by the research associate in cooperation with the parents. Motor impairment was classified using the GMFCS^30^; intellectual impairment as estimated IQ ≥ 70, 50–70 or <50. Intellectual impairment was assessed using an algorithm based on the questions “Do you think your child learns as well as other children of a similar age?”, “Are most of your child's friends a similar age to your child?”, “Does your child have severe difficulty learning in all aspects of development?”, “Do you think that your child needs much more help than other children to learn things like reading and understanding ideas?”

### 2.4 Data collection

Adolescents with CP were asked to complete the QYPP-SF questionnaire at home visits by a research associate. Most often the adolescent was alone with the research associate who provided help if needed; for example by reading the questions or ticking the boxes if a motor impairment made it difficult. If the adolescent was not able to complete due to intellectual impairment, the questionnaire was completed by a parent.

We regard frequency of participation as an objective measure of what adolescents do. In order to have the most accurate data, we used the report of the adolescent if he/she could self-report and of the parent if the adolescent could not self-report. Consequently, we analysed self-reported and proxy reported data together assuming this was the best estimate of participation.

In most regions the adolescents in general populations completed the questionnaire during a school lesson and no evaluation of impairment was performed.

### 2.5 Statistical methods

The QYPP has seven domains: *Getting on with other people, Autonomy, Recreation, Home life, Education, Work/finances and Preparing for the future*. In the QYPP short form, used in SPARCLE2, three of these domains (*Getting on with people, Recreation and Autonomy*) had more than three items and therefore could be examined for presence of latent traits. The domain Preparing for the future had only one item, “discuss when to live independently” which we added to the *Autonomy domain*. From the three remaining domains fewer items remained and consequently only analyses of single items were feasible (see below). We considered the Recreation domain qualitatively to consist of three sub-domains: *Community, Physical and Sedentary recreation* (see Table 3A and B), but only *Community recreation* had enough items to seek a latent trait. Responses were categorical but, since the intention was to measure the frequency of participation we transformed them into indicators of frequency per month and analysed them as continuous variables. Some response categories were not exact frequencies and we then estimated “mean frequency”. For example “most days but not every day” was translated into 22 in 30 days. Response categories in the domain of Autonomy were not frequencies and consequently not transformed into days per month (for complete of response categories see Table 3A). The item of “using a phone” was recoded to have a maximum of 60 (two times a day) and combined with the item of “online communication” into one variable defined as the highest score of the two items. Frequencies of different activities per month varied considerably and therefore all variables included in domains were standardised to a mean of 0 and standard deviation of 1. Checks on construct validity of the three domains were undertaken using confirmatory factor analysis and differential item functioning.

Single items were dichotomised into high and low frequency of participation according to the median in the total population of adolescents with CP and the general population. We analysed single items representing sub-domains of recreation: *Physical recreation* and *Sedentary recreation*, as well as the domains of *Home life* and *Educational life* using logistic regression models of binary outcomes of frequency of participation. We again included adolescents with and without CP and analysed by severity of impairment and region, while adjusting for age and gender. All logistic regression models were tested with Hosmer–Lemeshow Goodness of Fit to check for heterogeneity, a satisfactory fit being indicated by a non-significant result (*p* ≥ 0.05).

The data on formal and informal jobs (items from the *Work/finances domain*) were combined and analysed by simple frequencies, as was the item on watching TV.

Statistical analysis was carried out using SAS^®^ version 9.2.

### 2.6 Psychometric evaluation of domains

The three domains (*Getting on with people, Community recreation* and *Autonomy*) were analysed with confirmatory factor analysis. Chi-square measured fit as a function of the difference between expected and observed covariance; Root Mean Square Error of Approximation described the fit as a function of the residuals of the model; Bentler's Comparative Fit Index described the fit while allowing for the degrees of freedom in the model. Criteria for a satisfactory fit were: *p*-value from chi-square >0.05, RMSEA < 0.06, Bentler's Comparative Fit Index > 90. Domain scores were defined as sum of items scores weighted according to factor loadings. They were analysed using ANOVA to investigate differences between adolescents with and without CP by severity of impairment and region, while adjusting for age and gender. Adolescents without CP were assigned the level ‘no impairment’. We assessed interactions between severity of impairment and region at a 10% level, so as not to overlook interactions due to lack of statistical power.

Table 3A and B shows all analysed items and domains. The domains of *Autonomy* and *Getting on with people* had a satisfactory fit. However, the domain of *Community recreation* had a poor fit, with 125 adolescents (2.7%) from the general populations and 9 adolescents with CP (1.3%) fitting very poorly. These adolescents were distributed equally across all regions, ages and genders, and were characterised by often going shopping, to live music and/or eating out at restaurants most days or every day. By excluding these adolescents, the domain had a satisfactory fit.

The above three domains with satisfactory fit were tested for differential item functioning, to study whether the questions behaved differently in different regions, for different levels of impairment or in the general populations. In the domains of *Getting on with people* and *Community recreation*, there was little differential item functioning, while in the domain of *Autonomy* we found differential item functioning according to the interaction between region and severity.

## 3 Results

### 3.1 Frequency of participation captured by domains with latent trait

**Fig. 1:**
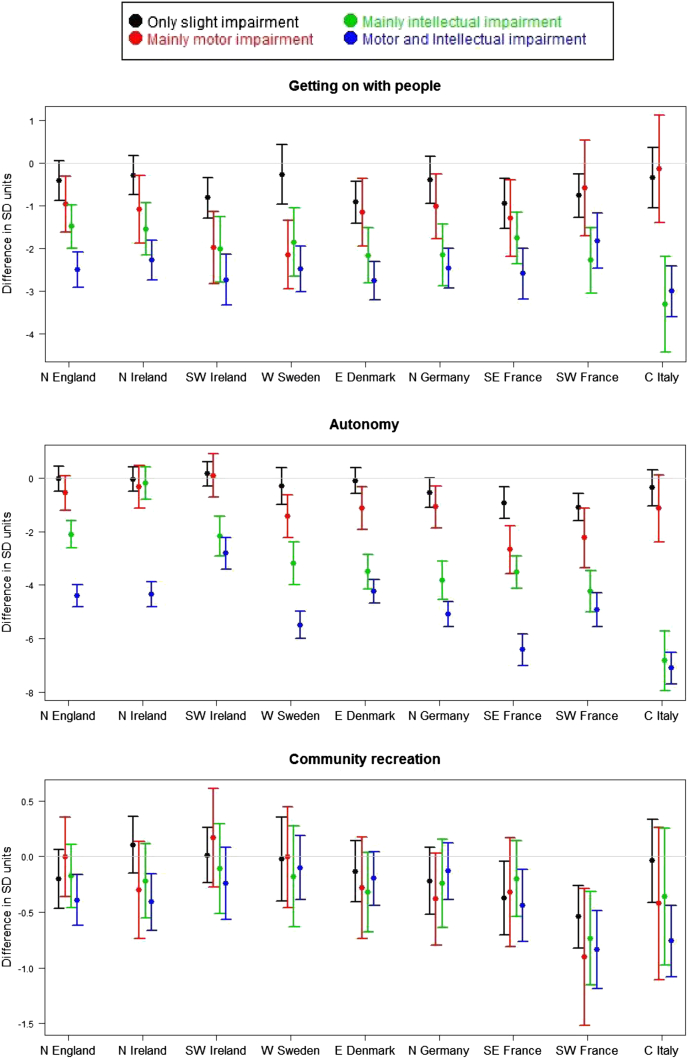
Comparison of participation of adolescents with CP and those in the general populations across the three domains with latent traits. Differences are in standard deviation units. Vertical bars show the 95% confidence interval of the difference. Difference >0 means adolescents with CP participate more often than adolescents in the general populations. Difference <0 means adolescents with CP participate less often than adolescents in the general populations.

Fig. 1 shows frequency of participation in the domains of *Getting on with people, Autonomy* and *Community recreation*, reported for each of the four groups of severity compared to adolescents in the general populations within each of the nine participating regions.

Adolescents with CP overall spent considerably less time and communicated less often with their friends than adolescents in the general populations (Fig. 1). A clear difference between groups of different severity was seen, but even adolescents with an only slight impairment were in general disadvantaged. Almost no regional differences were seen except that central Italian adolescents with intellectual impairment had very low participation compared to their able-bodied peers.

A similar effect of severity was seen for *Autonomy*, but with a larger disadvantage among adolescents with a combined intellectual and motor impairment. Most adolescents with an only slight impairment reported being as autonomous as adolescents in their general populations. However regional differences existed with adolescents in north England and the Irish regions generally scoring higher than those in the French regions and central Italy.

The domain of *Community recreation* showed a different pattern with little or no difference between adolescents with CP and the general populations and very small differences between the groups of different severity. Regional differences were small except that in southwest France adolescents with CP had lower participation than their able-bodied peers.

### 3.2 Frequency of participation captured by single items

For most single items the effect of type and severity varied with region and consequently results for each region are reported separately. But for “chores at least weekly” type and severity of impairment had a similar effect on frequency of participation in all regions and consequently adolescents in all regions were analysed together.

**Fig. 2:**
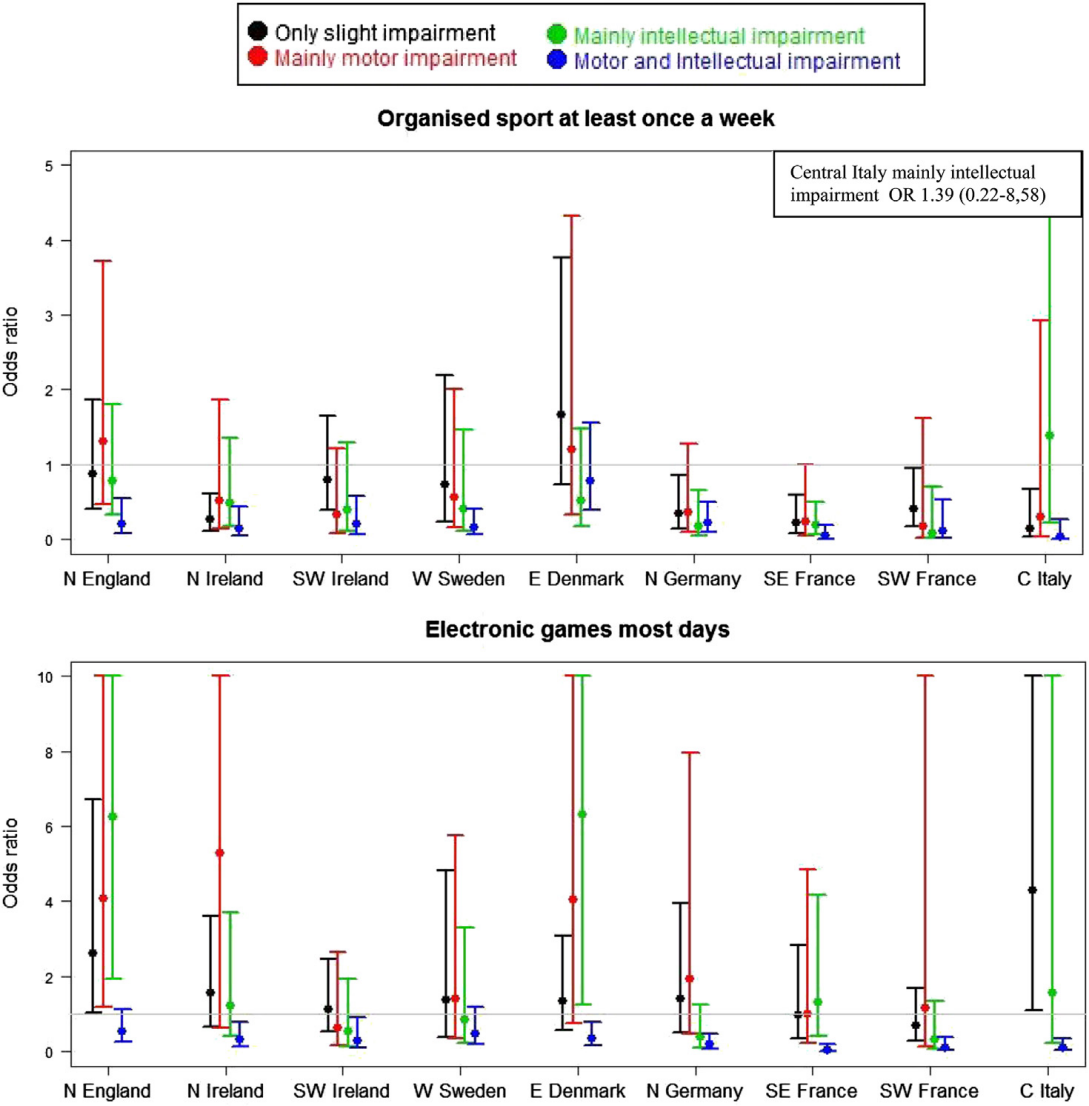
Comparison of participation of adolescents with CP and those in the general populations in single items of *Physical recreation* and *Sedentary recreation*. Odds ratios (ORs) compare participation of adolescents with CP and of those in the general populations according to region and severity of impairment. Vertical bars show the 95% confidence interval of the ORs. OR >1 means adolescents with CP participate more often than adolescents in the general populations. OR <1 means adolescents with CP participate less often than adolescents in the general populations. Confidence intervals of estimates of playing electronic games are cut at OR 10. All adolescents with a mainly motor impairment in Central Italy played electronic games most days and consequently no OR was calculated.

The single items *Physical recreation* (asking about ‘organised sport’) and *Sedentary recreation* (asking about ‘electronic games’ and ‘TV’) revealed different patterns (Fig. 2). Adolescents with CP engaged less often in ‘organised sport’ than adolescents in the general populations. Only minor regional variation was found but adolescents in east Denmark and north England were more likely than those in other regions to play organised sport as frequently as adolescents in the general populations. Adolescents with a combined motor and intellectual impairment were less likely to play electronic games daily than adolescents in the general populations. Adolescents with all other severities of CP in most centres were more likely to do so. In total 4% of adolescents with CP never watched TV, compared with 1% of adolescents in the general populations (not shown). Among those ever watching TV, a higher proportion of adolescents with CP did so daily (68%), compared with adolescents from the general populations (61%). No significant differences between groups of different severity were found, but regional differences existed. Adolescents (with CP and from the general populations) in north England, southwest Ireland and central Italy more often wachted TV daily compared with other regions.

**Fig. 3:**
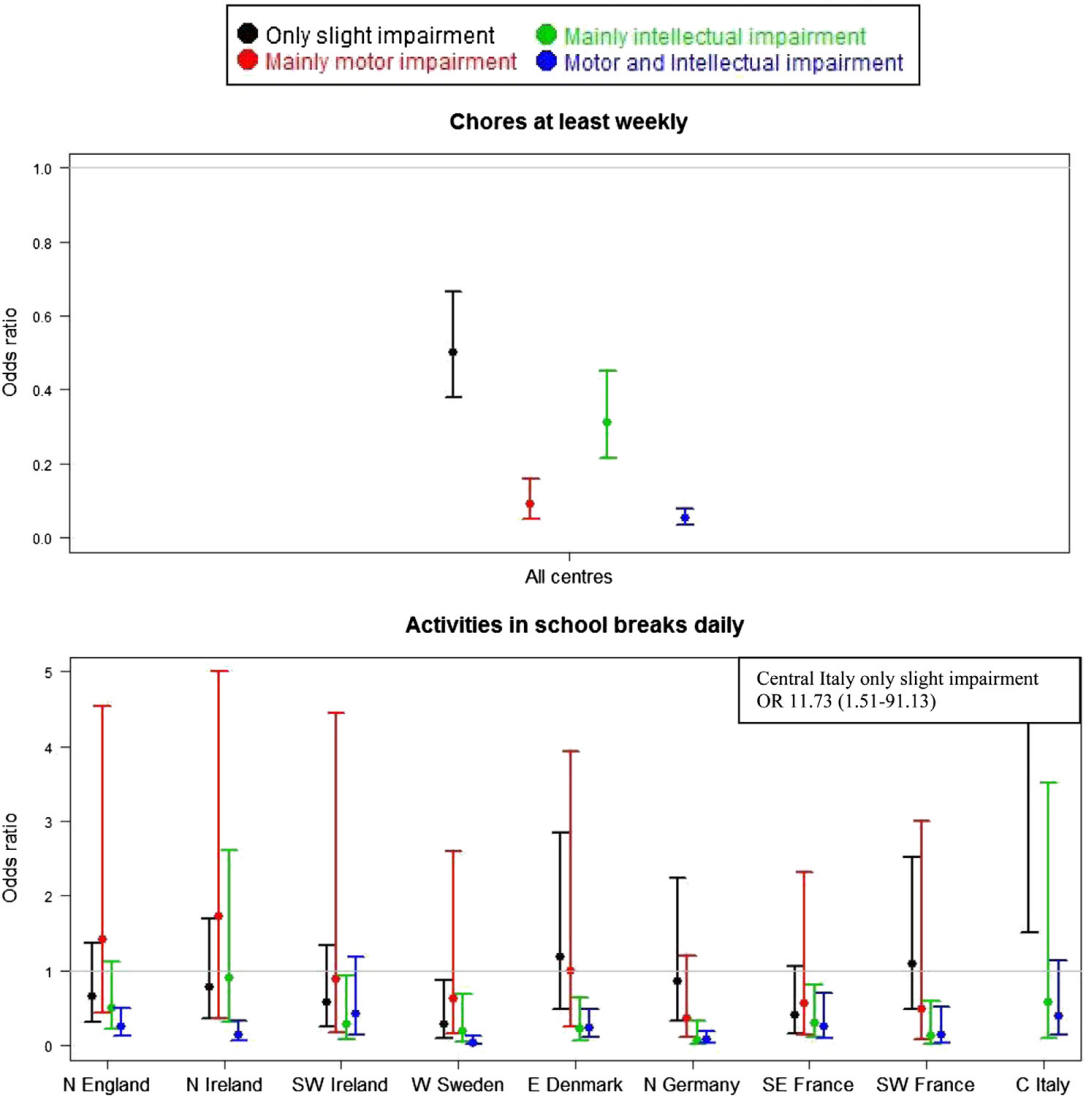
Comparison of participation of adolescents with CP and those in the general populations in single items of *Home life* and *Educational life*. Odds ratios (ORs) compare participation of adolescents with CP and those in the general populations according to region and severity of impairment. Vertical bars show the 95% confidence interval on the ORs. OR >1 means adolescents with CP participate more often than adolescents in the general populations. OR <1 means adolescents with CP participate less often than adolescents in the general populations. All adolescents with a mainly motor impairment from central Italy participated in activities in school breaks most days and consequently no OR was calculated.

Data for *Home life* (asking about ‘doing chores’) and *Educational life* (asking about ‘informal activities in school breaks’) are shown in Fig. 3. All adolescents with CP and especially adolescents with a motor impairment helped less often with chores at home than adolescents in the general population. Adolescents with an only slight or only motor impairment tended to participate as often in ‘informal activities in school breaks, while adolescents with an intellectual impairment in most centres did so considerably less often than adolescents in the general populations. Few regional differences existed except that central Italian adolescents with an only slight impairment had high participation compared to their able-bodied peers.

For *Work life* (asking about ‘formal and informal jobs’), 8% of adolescents with CP had a job compared to 44% of adolescents in the general populations (Table 3). In both groups an informal job was more prevalent than a formal job: 7% vs 3% for those with CP and 37% vs 20% for those in the general populations. Nearly all the adolescents with CP who were employed had an only slight impairment. We did not analyse this further due to the small numbers involved.

No single items showed heterogeneity according to Hosmer–Lemeshow Goodness of Fit.

### 3.3 Characteristics of responders and non-responders

Analyses of attrition between SPARCLE1 and 2 identified non-responding families to have higher levels of parental stress, lower educational qualifications and their adolescents to have milder motor impairment.^27,28^ Additional analyses for this paper showed that in most participation domains, frequency of participation in childhood (in SPARCLE1) did not differ between adolescents participating and not participating in SPARCLE2. We are not able to characterise non-responders in the general population.

## 4 Discussion

Compared to adolescents in the general population, adolescents with CP spent time less time with friends, played sports less often, led more sedentary lives and felt less autonomous in everyday life. Those with more severe impairment, especially intellectual impairment, had even less social contact and decisional autonomy. There was some regional variation.

The lower frequency of participation in adolescents with CP compared to the general population of the same age is consistent with other studies.^19–21,24^ The effect of motor impairment is also consistent with other studies,^24,31^ but we found no studies that investigated the effect of other impairments. Children with more severe motor impairment are more likely to have problems in communication and cognition and consequently difference in participation cannot be attributed only to motor impairment.^31^ Our study suggests that intellectual impairment may have a larger effect on participation than motor impairment, and therefore supports the call for inclusion of children with more complex disabilities, such as severe communicative and cognitive problems, in studies of participation.^20^

Most studies on frequency of participation in adolescents with CP used the well-validated instrument CAPE.^32^ We chose the new instrument of QYPP for this study since we needed a shorter instrument including specific adolescent activities like texting, online communication, spending time with friends without adult supervision and having a romantic relationship – such features are not part of the CAPE.

Autonomy is important for all people. Some people with severe impairment never achieve physical independence, but this should not prevent them from making independent decisions. Adolescents with CP in a recent Canadian study highly valued choosing their own activities.^33^ In our study, autonomy was captured a the extent to which the adolescents decided whom to spend their time with, how to plan their day, how to use their money and whether they took part in discussions on when to live by themselves. It is reassuring that adolescents with CP with an only slight impairment in most regions have similar autonomy to those in the general populations in the same region. Adolescents with more severe but mainly motor impairments in the English and Irish regions also have similar autonomy to the general populations; this is not so for the other regions and the reason for this is unclear.

Fewer adolescents with CP had work experience, compared with adolescents in the general populations. This is consistent with a US study where significantly fewer adolescents with spinal cord injuries had experience with paid work compared to their friends and siblings.^34^ Reasons suggested were that parents do not expect their child to become independent and employed.^34^ Work experience at a young age might facilitate later participation in the labour market. An earlier US study found work during high school in adolescents receiving special education predicted wages and percentage of time employed after graduation.^35^ The first step towards work experience could be assisting with household chores at home. In our study even adolescents with an only slight impairment did chores at home significantly less often than adolescents in the general populations and this was also evident in childhood.^6^ We can only speculate on the reasons for this and fatigue, pain, parental concessions and delayed development need to be considered.

For *Community recreation,* such as going on holiday and eating out, we found only minor disadvantages for adolescents with CP. The effect of severity of impairment was also small. This could be due to the rather crude measure of impairment which did not include, for example, the ability to communicate and feeding. However difficulties in communication and feeding are often associated with motor or intellectual disabilities. A high frequency of eating out and going on holidays among southwest French adolescents in the general population contributes to the large disadvantage found in such activities of southwest French adolescents with CP.

Our results regarding ‘organised sports’ are consistent with an Australian postal survey of 112 adolescents with CP who were found to be less physically active than their peers without impairment.^24^ The study did not find any differences in sedentary behaviour, defined as numbers of hours watching TV, playing computer or videogames.^24^ This is also in accord with our results but, taking type and level of impairment into account, we found most adolescents with CP played electronic games more frequently, while adolescents with a combined motor and intellectual impairment did so less often. The Australian study found no significant differences between groups described by level of gross motor function, but did not consider intellectual impairment. It is possible that adolescents with CP who are able to engage in this kind of active sedentary behaviour might prefer this to physical activities. A Dutch study using the CAPE questionnaire in 6–18 year old children also found significantly lower frequency of active physical activities in children with a physical disability compared with children without a disability.^20^

Frequency of participation was analysed in SPARCLE1 for the same adolescents when they were 8–12 years.^6^ Although a different instrument was used, some themes were the same. Children with CP participated less often in sports and outdoor games than children in the general populations except in eastern Denmark and north England. We found the same in adolescence indicating that patterns of participation established in childhood continue in adolescence. At age 8–12 years the children with a mild or moderate intellectual or motor impairment played computer games more often than children in the general population, also suggesting that little physical activity and frequent sedentary behaviour are problems which start before adolescence in children with CP. However these hypotheses need to be examined in longitudinal analyses.

Formal and informal contact with friends is important and valued by adolescents with and without disability. In a recent study of 12–20 year old young people with CP in Canada social activities were the most enjoyed.^33^ We found adolescents with CP less often meet friends without adult supervision, less often use virtual media and less often spend time with a boy/girlfriend. This is consistent with a Dutch study of young adults with CP aged 18–24 years^36^ which found a strong association between romantic relationships and participating in peer group activities. A small study of adolescents in special schools in Israel found those with CP were more likely to participate in activities at home and alone, while adolescents without disabilities more often engaged with friends.^19^

In our study, adolescents with an only slight impairment CP in some regions had nearly the same level of participation in the domain of *Getting on with people* as adolescents in their general populations. Reasons why adolescents with CP communicate less by virtual media could be that they are more often supervised by adults or that they have poorer motor skills.

Adolescents with intellectual impairment participated considerably less often in informal activities in school breaks, like chatting and relaxing with friends, than adolescents with only slight or mainly motor impairment. A qualitative British study suggests that constant adult supervision in schools prevents normal peer interaction.^37^ In our study it was mainly adolescents with intellectual impairment, a group often supervised by adults, who were restricted in these informal activities. In addition a Swedish study found that students over age 13 years experienced more unmet needs in environmental adjustments in mainstream schools than younger students.^38^ For example they often had to change classroom resulting in poor access to personal non-portable equipment; and more teachers needed to be made aware of their needs. The authors argue that increasing problems for adolescents may result from their environment becoming less accessible. Availability of a satisfactory social, physical and attitudinal environment is associated with higher participation in children with CP; specifically participation in school is influenced by the attitudes of teachers and helpers.^10^

### 4.1 Strengths and weaknesses of the study

SPARCLE is a large study based on random sampling from population-based registers. Some attrition occurred following recruitment at age 8–12 years; drop-out was not related to frequency of participation at age 8–12 years but we cannot rule out that adolescents with CP who did not take part might have biased the results. For the general population data, a larger proportion of schools did not take part in eastern Denmark and western Sweden; and response rates within a school were especially low in northwest Germany, probably due to completion not being allowed in school lessons. We do not know the characcteristics of those in the general populations who did not want take part in the survey. Theses issues could affect the representativeness of the populations.

Participation in this study was measured as frequency. More participation is not necessarily better; choice and enjoyment are also important. However we believe we gain a reliable estimate of achieved level of equity when we compare adolescents with and without CP in terms of frequency in the main areas of participation. There is no reason to think that a population of adolescents with CP in general should prefer a lower level of participation in for example social activities than other adolescents of the same age.

Although three domains of participation could be represented by valid latent traits, especially the domain of *Autonomy* showed differential item functioning. Consequently the items on decisional autonomy might not mean exactly the same for different groups of adolescents with CP and adolescents in the general populations in all regions. Finally the finding that the same level of severity of CP does not affect adolescents from different regions equally is interesting, but subgroups are of small size and results should be interpreted with caution.

### 4.2 Implications and conclusion

Most adolescents with CP participated considerably less often in social and physical activities and experienced less decisional autonomy than adolescents in the general population in the same region. Severity and type of impairment strongly predicted frequency of participation and especially children with intellectual impairment were disadvantaged.

Spending time and communicating with friends as well as reaching a higher level of independence are crucial for adolescents with and without CP, but seem to be a challenge for adolescents with CP across all European regions. However some regions did better than others. There has recently been an increasing focus on encouraging physical activity. Adolescents with CP and especially adolescents with an intellectual impairment seem to be at a higher risk of spending a lot of time on sedentary behaviour and less time on physical activity, compared to adolescents in the general populations. Some regions succeed better in engaging adolescents with CP in organised sports. Few adolescents with CP had job experience and this might reduce their opportunities for social contact and development of social skills as well as their chance of later employment.

Participation is an important health outcome; and participation can be influenced by personal and environmental factors. Personal and environmental predictors of participation of adolescents with cerebral palsy need to be identified in order to design interventions directed to such predictors; and in order to inform the content of services. There is also a need to investigate what might explain the regional differences in participation we have identified.

## Figures and Tables

**Table 3A:** Domains of frequency of participation.

Domains		Frequency of participation			Confirmatory analyses of domains			
		CP total	CP self-report	General population	RMSEA	CFI	*P*-value (Chi^2^)	Factor weights assigned
	Items included in domains	Median number of days participated per 30 days						
Getting on with people^a^	Time with friends without adults	1	4.3	22	0.0195	0.9981	0.0525 (5.90)	0.7065
	Spend time with boy/girlfriend	0	0	0				0.3830
	Go to friends' houses to hang out	0	2.5	4.3				0.5716
	Use of phone or online (two items combined)	22	30	30				0.4324
Community recreation^b^^,^^c^	Go shopping for pleasure	1	2.5	2.5	0.0475	0.9760	<0.0001 (25.87)	0.4279
	Eat meals at café/restaurant	1	2.5	2.5				0.5788
	Go to live music events	0.4	0.4	0.4				0.4748
	Go on holiday	0.1	0.1	0.2				0.2054
Median score								
*Autonomy*	Decide own daily routine	Sometimes	Mostly	Almost always	0.0046	0.9999	0.3287 (2.22)	0.6105
	Decide how to spend money	Mostly	Mostly	Almost always				0.7165
	Choose who to spend time with	Mostly	Almost always	Almost always				0.7817
	Discuss when to live independently	Never	Never	Max. every 2–3 month				0.1440

a Text from response categories and corresponding score: Every day = 30, Most days = 22, One a week or less = 4.3, 2–3 times per month = 2.3, Once month = 1 and Never = 0.

b Text from response categories and corresponding score: 2–3 times per month = 2.3, Once month = 1, Max. every 2–3 months = 0.4, Twice a year and = 0.2 Once a year = 0.1.

c Excluding 125 from the general populations and 9 with CP with a very high frequency of participation in recreational activities (see Method section).

**Table 3B:** Single items of frequency of participation.

	Analyses of single items	Frequency defined as low participation	Frequency defined as high participation	Percentage of adolescents with high participation		
Sedentary recreation	Play electronic games	Once a week or more seldom	Most days	52	64	52
	Watch TV	Most days or more seldom	Every day	65	68	60
Physical recreation	Organised sport	2–3 times a month or more seldom	At least once a week	33	41	55
Home life	Do chores	2–3 times a month or more seldom	At least once a week	32	42	70
Educational life	Informal activities in school breaks	Most days or more seldom	At least daily	49	60	70
Work life	Having a job (two items combined)	Currently not having a formal or informal job	Currently having a formal or informal job	8	12	44

**Table 1:** Characteristics of adolescents with CP in nine regions.

	N England	N Ireland	SW Ireland	W Sweden	E Denmark	NW Germany	SE France	SW France	Central Italy	All regions
***N* children (% self-reporting)**	109 (68%)	88 (74%)	77 (74%)	68 (60%)	86 (67%)	74 (70%)	65 (71%)	58 (67%)	42 (43%)	667 (67%)
**Mean age (range)**	15 (12–18)	14 (12–18)	15 (12–18)	15 (12–17)	15 (12–18)	14 (12–18)	14 (12–17)	14 (11–18)	15 (12–18)	15 (12–18)
	%	%	%	%	%	%	%	%	%	%
**Age group**										
11–13y	29	30	25	12	19	43	35	42	17	28
14–15y	39	45	45	54	37	30	43	28	40	40
16–18y	32	25	30	34	44	27	22	31	43	32
**Gender (% male)**	58	58	53	57	52	58	58	67	52	57
**Motor function GMFCS**^a^										
I	35	25	40	29	41	28	38	43	29	34
II	16	30	22	9	8	16	20	21	17	18
III	20	10	9	4	13	20	11	10	14	13
IV	14	18	10	18	12	15	11	10	14	14
V	15	17	18	40	27	20	20	16	26	21
**Intellectual function**										
IQ ≥ 70	42	47	60	39	43	43	42	53	43	46
IQ 50–70	30	30	21	28	36	19	33	16	7	26
IQ < 50	28	24	19	33	21	38	25	31	50	28
**Impairment**										
Only slight impairment GMFCS I or II AND IQ ≥ 70	28	35	47	22	31	28	30	45	33	33
Mainly motor impairment GMFCS III, IV or V AND IQ ≥ 70	14	11	13	16	12	15	13	9	10	13
Mainly intellectual impairment GMFCS I or II AND IQ < 70	23	19	16	16	17	16	28	19	12	19
Motor and intellectual impairment GMFCS III, IV or V AND IQ < 70	35	34	25	45	40	41	30	28	45	35

a GMFCS Gross Motor Function Classification System.

**Table 2:** Characteristics and recruitment of adolescents in general populations in the nine regions.

	N England	N Ireland	SW Ireland	W Sweden	E Denmark	NW Germany	SE France	SW France	Central Italy	All regions
**Schools recruitment**										
Sampled among all schools in the uptake area	Yes	Yes	Yes	Yes	Yes	Yes except for no private schools	Yes	Yes except for fewer private schools	Yes except for no private schools	
Number of schools randomly selected from lists of schools (*N* selected/*N* listed)	6/158	10/219	6/111	10/1209	35/1001	15/221	10/281	6/125	7/171	
**Response rates**										
Response rate schools	83% (5/6)	80% (8/10)	50% (3/6)	40% (4/10)	37% (13/35)	80% (12/15)	70% (7/10)	100% (6/6)	100% (7/7)	62% (65/105)
Children enrolled	1195	1028	325	Unknown	1606	Unknown	386	Unknown	466	
Children present	1019	Unknown	249	Unknown	1272	2262	317	Unknown	406	
Children completing questionnaires	780	748	249	157	1247	1021	305	316	370	5193
Response rate students enrolled	65%	73%	76%	Unknown	78%	Unknown	79%	Unknown	79%	74%
Response rate students present	77%	Unknown	100%	Unknown	98%	45%	96%	Unknown	91%	72%
Percentage of valid questionnaires	81%	96%	93%	68%	87%	95%	98%	90%	91%	90%
**Adolescents included**	635	721	232	107	1081	972	298	283	337	4666
Age (mean, range)	14 (12–18)	15 (12–18)	15 (12–18)	15 (12–18)	15 (12–18)	14 (12–18)	14 (12–18)	14 (11–18)	15 (12–18)	15 (11–18)
Age group										
11–13y	28	30	22	23	25	42	29	35	19	30
14–15y	55	36	40	64	34	41	43	38	39	41
16–18y	17	34	38	13	41	16	28	27	42	29
Gender (% males)	47	54	47	56	48	43	39	38	55	47
